# Familial Disseminated Comedones Without Dyskeratosis: A Case Report

**DOI:** 10.7759/cureus.85647

**Published:** 2025-06-09

**Authors:** Ilse Marilu Gutierrez Villarreal, Circe Ancona Castro, Ivan E Cano Lizarraga, Monica Ceballos-Pérez, Grecia Mariana Cantu Fonseca, Diego E Gómez López, Carlos S Saenz de Leon

**Affiliations:** 1 Dermatology, Institute for Security and Social Services for State Workers (ISSSTE) Monterrey Regional Hospital, Monterrey, MEX; 2 Internal Medicine, Regional Hospital of High Specialty of the Yucatan Peninsula, Mérida, MEX; 3 Infectious Disease, Institute for Security and Social Services for State Workers (ISSSTE) Monterrey Regional Hospital, Monterrey, MEX

**Keywords:** disseminated familial comedones, genodermatosis, lamellar keratinization, non-dyskeratotic comedones, pen-2 mutation

## Abstract

Disseminated familial comedones without dyskeratosis (DFCWD) is a rare autosomal dominant genodermatosis characterized by widespread comedonal eruptions, predominantly affecting the trunk and face, in the absence of histopathological features such as dyskeratosis or acantholysis. While it exhibits clinical overlap with other entities within the spectrum of familial comedonal disorders, its unique histological features and inheritance pattern support its classification as a distinct clinical entity. Due to the limited number of reported cases, its phenotypic spectrum, natural history, and underlying molecular mechanisms remain poorly understood.

## Introduction

Disseminated familial comedones without dyskeratosis (DFCWD) is an autosomal dominant genodermatosis characterized by widespread and extensive comedonal involvement of the skin, distinguished by specific histopathological findings such as dilated follicles filled with laminar keratin and filiform extensions of basaloid cells in the outer root sheath, without evidence of acantholysis or dyskeratosis. It is a very rare dermatosis, with only a few families previously reported in the literature [1-3]. We describe the case of a 39-year-old woman who began experiencing symptoms at age 14, including comedones, painful nodules, scarring, and histopathological findings consistent with DFCWD. Similar clinical features were observed in family members, prompting their clinical evaluation.

## Case presentation

**Figure 1:**
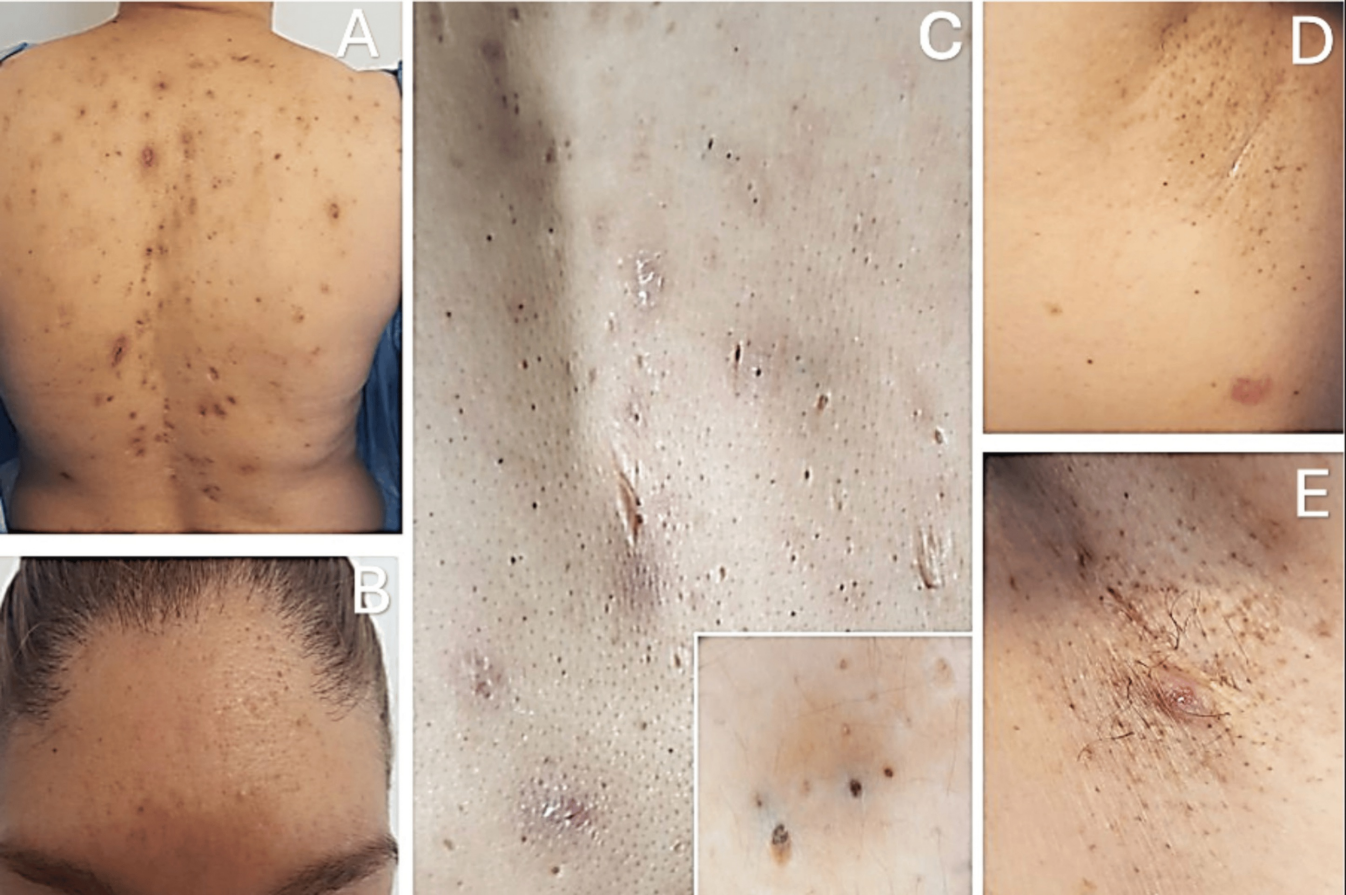
Multiple comedones and nodules on the face (A) and trunk (B). Numerous punctate depressions, along with some hypertrophic and retractile scars on the back (C). Few lesions observed in the armpits (D) and groin (E).

We report the case of a 39-year-old woman who presented to our clinic with a history of multiple comedones and papulonodular lesions beginning at 14 years of age. Upon physical examination, a polymorphic dermatosis was observed, distributed over the frontal region of the face, back, and axillary and inguinal folds. It consisted of numerous comedones (1-4 mm in diameter), most of which were covered by a dark brown central keratotic plug; multiple erythematous-violaceous nodules (1-3 cm) with follicular openings and keratotic plugs on their surface, some covered by blood crusts; a few hypertrophic and retractile scars; numerous pinpoint depressions; and multiple post-inflammatory brown macules (Figure 1).

**Figure 2:**
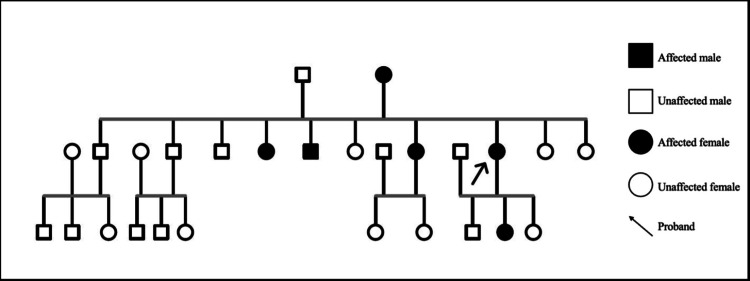
Pedigree chart of the patient (proband indicated by an arrow).

Notably, the dermatosis showed a clear predominance on the back with minimal involvement in the folds. The comedones were asymptomatic, while the nodules were painful. The dermatosis had been persistent since its onset at puberty, with a progressive increase in the number of comedones and intermittent formation of nodules. The remainder of the physical examination was unremarkable, with no relevant personal medical history, significant medication or chemical exposure, and no abnormalities in laboratory test results. Clinical investigation revealed a family history of similar skin lesions across three generations, with onset during adolescence and varying severity, affecting her mother, two sisters, a brother, and one of her daughters (Figure 2).

**Figure 3:**
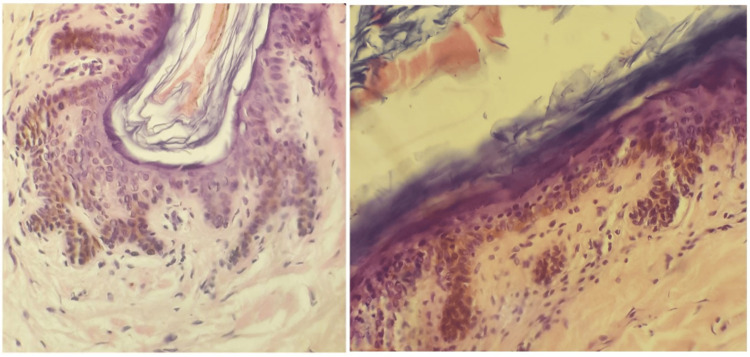
Dilated hair follicle filled with lamellar keratin, with a wall of multilayered epithelium showing arboriform extensions of basaloid cells, without dyskeratosis or acantholysis. Stained with H&E.

Histopathological examination of the lesions showed follicular dilations filled with lamellar keratin, with walls composed of stratified squamous epithelium and arborizing projections of basaloid cells in the external root sheath, without evidence of dyskeratosis or acantholysis (Figure 3). Genetic testing was deferred due to limited resources in our setting. The patient was started on oral isotretinoin at a dose of 0.5 mg/kg/day for twelve months. Improvement in the nodular lesions was noted by the ninth month; however, no significant changes were observed in the comedones.

## Discussion

Two rare dermatologic conditions are characterized by the distinctive clinical presentation of numerous disseminated comedones with onset during childhood or adolescence, often affecting several members of the same family with variable severity. The more extensively documented of these is known as familial dyskeratotic comedones, defined by its characteristic histopathological features: dilated follicles filled with keratin and lined by squamous epithelium exhibiting acantholysis and dyskeratosis [1, 2]. This entity is well recognized and classified as an autosomal dominant genodermatosis. The second, considerably rarer condition, is histopathologically distinguished by dilated hair follicles containing keratin plugs and arborizing projections of basaloid cells arising from the outer root sheath, in the absence of acantholysis or dyskeratosis. Referred to as DFCWD, this variant remains poorly understood due to the very limited number of reported cases, and its familial patterns and clinical characteristics have not yet been fully elucidated [3, 4].

In this case, the evaluation of a patient with disseminated comedones, without other manifestations, revealed a familial form of the dermatosis. Six individuals of both sexes across three generations were affected, suggesting autosomal dominant inheritance. Clinical and histopathological features aligned with previous reports: adolescent onset, predominant trunk involvement, variable severity, chronic course with poor treatment response, and biopsy showing crateriform follicular dilations with lamellar keratin [5]. Notably, the follicular walls exhibited branching basaloid cells, without acantholysis or dyskeratosis.

Given the limited number of reported DFCWD cases, the familial pattern and overall clinical characteristics of the disease remain undefined. Furthermore, it is not yet well established whether DFCWD constitutes a separate entity within the spectrum of familial comedonal disorders [3, 4]. A PubMed literature search identified three publications reporting cases of individuals with DFCWD, all demonstrating familial presentation with autosomal dominant inheritance and a wide range of severity, involving a total of 67 individuals across four families [3, 4, 6].

A genetic study of two families with DFCWD identified a heterozygous mutation in the PEN-2 gene on chromosome 19, linked to increased PEN-2 expression in leukocytes [7]. While the effects of this increase are yet to be fully evaluated, it is thought to be the primary contributing factor. PEN-2, a subunit of the γ-secretase protease complex, cleaves transmembrane proteins such as amyloid precursor protein and Notch receptors. Mutations in genes encoding γ-secretase subunits have been implicated in diseases such as familial Alzheimer's disease, dilated cardiomyopathy, breast cancer, leukemia, and hidradenitis suppurativa [7, 8].

To establish an accurate diagnosis, a thorough evaluation of additional clinical information, such as age of onset, associated signs and symptoms, and histopathological findings, was essential to exclude other differential diagnoses. These included: familial comedones with dyskeratosis, excluded based on the absence of dyskeratotic changes on histopathology; chloracne, ruled out due to a negative history of exposure to chlorinated compounds such as pesticides or herbicides; Darier’s disease, dismissed due to the lack of characteristic keratotic papules on seborrheic or sun-exposed areas; Dowling-Degos disease, excluded based on the absence of reticulated hyperpigmentation in flexural regions; and hidradenitis suppurativa, excluded due to limited axillary and inguinal involvement, absence of typical anatomical distribution (perianal, perineal, mammary, submammary), predominance of comedonal lesions on the back, and lack of characteristic histopathological features such as perifolliculitis, destruction of pilosebaceous units, or granulation tissue.

Considering the evidence presented, we propose that this case represents a typical presentation of DFCWD, a condition of clinical relevance due to its rarity. The genealogical analysis supports an autosomal dominant inheritance pattern. Continued follow-up of the family is essential for further characterization of the condition, particularly as two affected individuals have not yet had offspring, and three members of the third generation are currently between 6 and 15 years of age, raising the possibility of future development of the characteristic cutaneous manifestations. Treatment is complex due to the lack of an effective therapy [2, 9]; however, oral isotretinoin has been shown to reduce morbidity by controlling inflammatory lesions and associated symptoms. Long-term surveillance is important given the reported association with squamous neoplasms in previous reports.

## Conclusions

This case reinforces the recognition of disseminated familial comedones without dyskeratosis as a distinct clinical and histopathological entity within the spectrum of inherited genodermatoses. The consistent clinical presentation across multiple generations, along with the characteristic histopathological findings and the apparent autosomal dominant inheritance pattern, supports its classification as a unique disorder. While oral isotretinoin has demonstrated partial efficacy, particularly in controlling inflammatory lesions, no curative therapy currently exists. Genetic counseling should be considered for affected families, although limitations in local infrastructure may pose challenges to its implementation. Comprehensive clinical documentation, long-term dermatological follow-up, and advanced genetic analysis remain essential for further elucidating the pathogenesis, refining diagnostic criteria, and identifying potential therapeutic targets for this rare genodermatosis.
